# Artemether-lumefantrine versus artesunate plus amodiaquine for treating uncomplicated childhood malaria in Nigeria: randomized controlled trial

**DOI:** 10.1186/1475-2875-5-43

**Published:** 2006-05-16

**Authors:** Martin Meremikwu, Ambrose Alaribe, Regina Ejemot, Angela Oyo-Ita, John Ekenjoku, Chukwuemeka Nwachukwu, Donald Ordu, Emmanuel Ezedinachi

**Affiliations:** 1 Calabar Institute of Tropical Disease Research & Prevention, GPO Box 1211, Calabar, Nigeria; 2 Federal Ministry of Health, Abuja, Nigeria

## Abstract

**Background:**

The therapeutic efficacy of artesunate plus amodiaquine and artemether/lumefantrine were assessed in an area of Nigeria with high levels of *Plasmodium falciparum *resistance to chloroquine and sulphadoxine-pyrimethamine.

**Participants:**

Children aged 6 to 59 months with uncomplicated *P. falciparum *infection and parasite density 1,000 to 200,000 parasites/μL enrolled following informed consent by parents.

**Methods:**

Eligible children were randomly assigned to receive either a 3-day course of artesunate (4 mg/kg) plus amodiaquine (10 mg/kg) or 6-dose course of artemether/lumefantrine (20/120 mg tablets) over three days. Patients were followed up with clinical and laboratory assessments until day 14 using standard WHO in-vivo antimalarial drug test protocol.

**Results:**

A total 119 eligible children were enrolled but 111 completed the study. Adequate clinical and parasitological response (ACPR) was 47 (87.0%) and 47 (82.5%) for artemether-lumefantrine (AL) and artesunate+amodiaquine (AAMQ) respectively (OR 0.7, 95% confidence interval 0.22 to 2.22). Early treatment failure (ETF) occurred in one participant (1.8%) treated with AAQ but in none of those with AL. Two (3.7%) patients in the AL group and none in the AAQ group had late clinical failure. Late parasitological failure was observed in 9 (15.8) and 5 (9.3%) of patients treated with AAQ and AL respectively. None of participants had a serious adverse event.

**Conclusion:**

Artemether-lumenfantrine and artesunate plus amodiaquine have high and comparable cure rates and tolerability among under-five children in Calabar, Nigeria.

## Background

Malaria accounts for more than one million deaths of mostly African children yearly. Early detection and prompt treatment is a key component of the global strategy for malaria control [1]. Across Africa, *Plasmodium falciparum *resistance to common affordable antimalarial drugs, chloroquine and sulphadoxine-pyrimethamine has reached very high levels, and noticably hampered malaria control efforts in the region [2]. In Nigeria, chloroquine and sulphadoxine-pyrimethamine have been the first and second line anti-malaria drugs respectively but evidence from local research spanning a period of two decades show that the therapeutic efficacy of these two drugs have deteriorated due to high levels *of P. falciparum *resistance in all parts of the country [3-5].

The World Health Organization recommends that combination treatment rather than monotherapy should be used in areas where multi-drug resistance to *P. falciparum *is a problem [6]. Combination drugs act at different sites or have different mechanisms of action, achieve better cure rates and are more likely to delay development of parasite resistance [7,8]. Artemisinin-based combination treatments (ACTs) are preferred because artemisinin compounds have rapid parasite and fever clearance effects and also reduce gametocyte rate with the potential to reduce transmission. Artemisinin derivatives have short half life but the component combination drug, with a longer duration of action, makes up for this by providing sustained activity to eliminate remaining parasites[7].

The artemisinin-based combination treatment regimens are more expensive than each drug used singly, but their advantages over monotherapy far outweigh the cost. Artemisinin-based combination treatments are recommended for use as first-line treatment for uncomplicated malaria, even in resource-poor areas where multi-drug resistant *P. falciparum *infection is a problem [6]. ACTs have been used for a long time as first-line treatment in some South-East Asian countries [9,10].

The options under consideration in Nigeria for ACTs are amodiaquine with artesunate, or artemether-lumefantrine. Artemether-lumefantrine has reportedly high cure rates in observational studies, but controlled trials comparing it against other artemisinin containing regimens are few [11]. It has an advantage that the component drugs are coformulated in the same tablet but it is expensive, even at discounted rates, and requires patients to take six doses. Amodiaquine plus artesunate is less expensive, but not co-formulated, and therefore more likely to be misused as monotherapy. It is believed that cure rates of this combination regimen may be lower than with artemether-lumefantrine because of parasite resistance to amodiaquine.

The Nigerian government supported randomized controlled trials in the six geopolitical zones of the country to assess the clinical efficacy and parasitological response of *P. falciparum *to combinations of artemether/lumefantrine and artesunate plus amodiaquine in treating uncomplicated childhood malaria. This paper describes one of these trials, conducted in the far-south geo-political zone within the Niger Delta region of the country. *P. falciparum *resistance to chloroquine was first documented in this region in 1987 and the current rate of resistance to chloroquine and sulphadoxine-pyrimethamine (SP) exceeds 80% [3,12].

## Methods

### Study area and population

This study was conducted in Ikot Ansa a rural community within the Calabar Municipal Local Council area of Cross River State, Nigeria. The area has a projected population of 222,100, with 44,420 being under five years of age. This area lies within the tropical rainforest belt of southeastern Nigeria. The annual rainfall is 2,000–3,000 mm, within the rainy season lasting from April to October. There are two predominant ethnic groups, *Efiks *and *Ejagham*. The people's main occupations are subsistence farming, fishing and logging. Malaria is holoendemic in the study area with high and perennial transmission, especially in the rainy season (from April to November). The study was conducted in the months of September, October and November, 2004.

### Patient enrolment

Children were enrolled if they had uncomplicated malaria, and fulfilled the following inclusion criteria: residence in study area, age 6–59 months, axillary temperature ≤37.5°C and parasite density of 1,000–200,000 asexual parasites/μl of blood, and informed consent by parents/guardian. Children with packed cell volume (PCV) <15%, severe malnutrition, other signs of severe malaria or illness including any "danger sign" (viz. inability to drink or breastfeed, vomiting everything, recent history of convulsion, lethargy or unconsciousness, inability to sit or stand up, were excluded from the study [12].

Ethical approval was given by the University of Calabar Teaching Hospital Ethical Committee. A written informed consent was obtained from each parent/legal guardian of eligible participants prior to enrolment.

### Randomization

Participants were randomized by simple random sampling technique (drawing lots) in blocks of ten (10) to receive either artemether-lumefantrine (AL) or artesunate+amodiaquine. Allocation sequence was concealed in opaque, sealed and serially numbered envelopes. Study treatment was started on the day of randomization (day zero) and completed on day two, while follow-up continued for 14 days. Clinical examination plus body weight and height were recorded on day zero. Follow-up visits were scheduled on days 1, 2, 3, 7 and 14. On each visit, clinical examination including axillary temperature and thick blood film specimen obtained to screen for presence of malaria parasites and density was assessed by light microscopy. Packed cell volume (PCV) was estimated on day zero and repeated on day 14.

### Interventions

Artesunate and amodiaquine were given orally at 4 mg/kg and 10 mg/kg body weight respectively, once daily on days 0, 1 and 2 with amodiaquine reduced to 5 mg/kg on day 2. Artemether/lumefantrine was given as 20/120 mg tablets. Participants weighing 5–14 kg received one tablet (20/120 mg), two tablets (40/240 mg) for body weight 1524 kg and three tablets for those weighing 25 – 35 kg. Each participant had a total of six doses of AL, with each dose at 12-hourly intervals for 3 days. Treatment was directly supervised by the research nurse and patients observed for 30 minutes. Drugs were re-administered to those who vomited within this period. Tepid sponging, exposure and administration of paracetamol at 15 mg/kg body weight were used to reduce high fever. Participants that did not return on schedule for follow-up were visited at home on the same day.

### Laboratory investigation

Thick and thin blood smears were prepared and stained in 3% Giemsa solution for 30 minutes. The smears were read to 100 fields with quantification of *P. falciparum *asexual parasitaemia on the thick smear. Parasites were enumerated using thick film as described by Shute [14]. The parasite density (per μl of blood) was calculated, assuming a normal leucocyte level of 8,000/μl. The thin film was used to speciate the parasites. Packed Cell Volume (PCV) was measured on days 0 and 14 with sample collected in a heparinized capillary tube and centrifuged for 5 minutes at 10,000 G.

### Analysis

Data generated were recorded in a log book, and individual patients case record files. Data were entered and analyzed with EPI-Info version 6.4. Data were also exported to SPSS version 11.0 for further analysis. Differences between groups were assessed using chi-square (X^2^), 't' test for continuous normally distributed variables and nonparametric Kruskal-Wallis was used to compare continuous not normally distributed variables.

**Figure 1:**
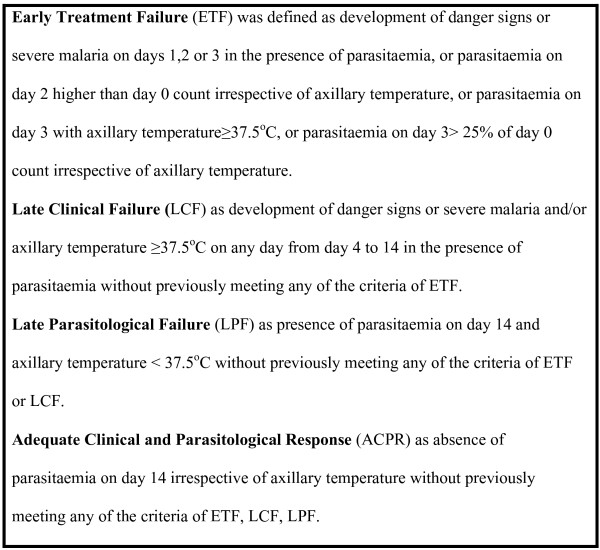
Classification of malaria treatment outcome.

Therapeutic efficacy was determined on day 14 or earlier by assessing both clinical and parasitological response based on the WHO guidelines for assessing therapeutic efficacy [15]. The classification of treatment based on this protocol is shown on Figure 1.

## Results

### Patients

**Figure 2:**
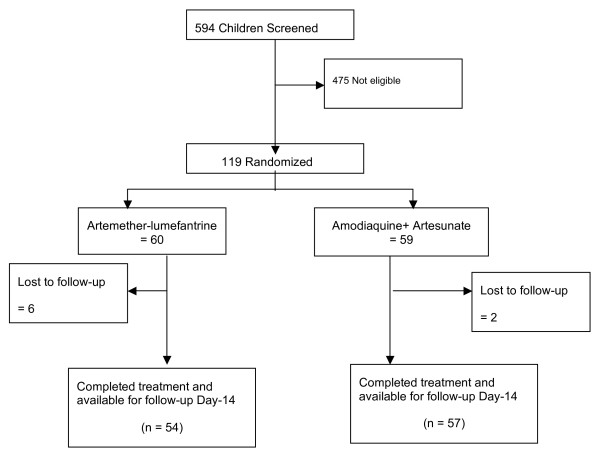
Trial Profile.

A total of 594 children were screened and 119 eligible ones were enrolled. The trial profile (Figure 2) shows the pattern and reasons for exclusion and losses to follow up. A total of six (10.0%) and two (3.4%) were respectively lost to follow-up in the artesunate/lumefandtrine (AL) and the artesunate/amodiaquine (AAMQ) groups. The AL arm had 60 participants, while the AAMQ arm had 59. A total of 54 participants who received AL and 57 who received AAMQ completed the trial, and had adequate data for the analysis of the end-points. Table 1 shows the baseline characteristics of the eligible participants. The two treatment groups were comparable in all characteristics. Data on previous antimalarial drug-use pattern shows that 11 (9.2%) of the enrolled participants had used antimalarial drugs in the past two weeks. The majority of these (8/11; 72.7%) had used chloroquine.

**Table 1:** Baseline characteristics of participants

Characteristics Mean (SD)	Artesunate + amodiaquine (N = 59)	Artemether-lumefantrine (N = 60)	Test statistics*
Age in months	29.1 (17.1)	28.2(16.2)	0.43†
Sex (Male) ‡:	28 (47.5)	33 (55.0)	0.68‡
Weight in Kg	12.4(3.8)	12.3(3.0)	0.59†
Height in cm (SD)	86.7(13.7)	85.6(12.7)	0.64†
Packed cell volume Day-0	28.8 (4.9)	28.9 (4.5)	0.32†
Packed cell volume Day-14	33.3 (3.6)	33.6 (4.0)	0.43†
Axillary temperature (°C)	38.5 (0.96)	38.6(0.81)	0.87†
Geometric mean parasite density in μL(range)	7972.0 (1,000 to 258,824)	7972.3 (1,011 to 318,769)	0.73§

^‡^Frequency (%) and Chi-squared test; ^†^t-test; ^§^Kruskal-Wallis H test;

*All tests show that both study groups were not statistically significant different.

### Outcomes

Table 2 shows the results of the 14-day therapeutic efficacy of the drugs. The number of evaluable participants with adequate clinical and parasitological response (ACPR) were 47 (87.0%) and 47 (82.5%) for AL and AAMQ, respectively. The difference in the cures rates of the two regimens was not statistically significant (Odds ratio 0.7, 95% confidence interval 0.22 to 2.22). Early treatment failure (ETF) was observed in one participant (1.8%) that received AAMQ but in none of those treated with AL.

**Table 2:** Therapeutic efficacy of artemether-lumefantrine *versus *artesunate+amodiaquine in Calabar Nigeria.

Indicators of treatment outcome	Number (%) of participants	
	
	Artesunate + Amodiaquine (n = 57)	Artemether-lumefantrine (n = 54)
Adequate Clinical and parasitological response (ACPR)	47 (82.5)	47 (87.0)
Early treatment failure (ETF)	1(1.8)	0 (0.0)
Late clinical failure (LCF)	0 (0.0)	2 (3.7)
Late parasitological failure (LPF)	9(15.8)	5 (9.3)

**Figure 3:**
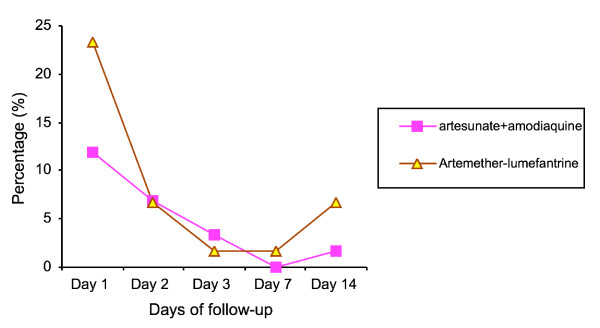
Percentage of children with fever (temperature > 37.4°C) during follow-up

Figure 3 shows that both treatment regimens had rapid and comparable fever clearance rates. One participant that received AAMQ had pains in the ear, which resolved before day 14. No other adverse events were observed in either treatment groups.

## Discussion

The key goal of artemisinin-based combination treatment (ACT) is to enhance cure rates and delay development of parasite resistance to component drugs [16]. This study has shown rapid parasite and fever clearance in children treated with 6-dose regimen of artemether-lumefantrine (AL) and 3-day course of artesunate plus amodiaquine (AAMQ). This is characteristic of artemisinin combination therapy [6]. It was also demonstrated that these ACT regimens were tolerable among under-five children in this locality. The ACTs were not compared with existing first-line and second-line drugs for uncomplicated malaria (chloroquine or sulphadoxine-pyrimethamine,) because of very high treatment failures (in excess of 80%) recently reported for both drugs in the study area [12]. Administering such ineffective treatments to ill, under-five children could significantly increase the risk of poor treatment outcome.

The cure rates obtained for a 6-dose regimen of AL and a 3-day course of AAMQ were high (> 80%) but fell short of therapeutic efficacy levels reported in other areas. Day-14 cure rates for AAMQ were 91%, 93%, and 98% in Kenya, Senegal and Gabon, with lower day-28 cure rates of 68%, 82% and 85%, respectively [17]. Another report of 14-day in-vivo study of AAMQ in Mozambique showed a parasitological cure rate of 94.3% [8].

The 14-day cure rate obtained for a 6-dose regimen of AL in the present study is comparable with those reported for 4-dose regimen in other trials, but lower than cure rates obtained in other 6-dose trials [9,11]. A 42-day cure rate of 93.6% has been documented in a recent randomized controlled trial of 6-dose regimen of AL [18].

This study has demonstrated comparable therapeutic efficacy and tolerability for a 6 dose regimen of artemether-lumefantrine and a 3-day course of artesunate plus amodiaquine. This apparent lack of difference in effects may be because the study samples were small and lacked statistical power to detect significant difference at 95% confidence level.

The short follow-up period of 14 days is another methodological limitation of this study. Recent observations have shown that treatment failures affecting artemisininbased combination treatment regimens tend to occur after 21 days [19]. The minimum follow-up period currently recommended this type of studies is 28 days. This trial started before the evidence that 14-day follow-up period is inadequate for in-vivo efficacy studies of ACTs became overwhelming.

The Nigerian government has reviewed the malaria treatment policy, replacing chloroquine and sulphadoxine-pyrimethamine with artemisinin-based combination treatment (ACT) as the first-line treatment for uncomplicated malaria. This change in policy has been informed by evidence from local studies like the one reported here [20]. Larger effectiveness studies with higher statistical power than the present study will be needed to ascertain the effects of these drug combinations in this area. Prospective sentinel monitoring of the use and effectiveness of the new ACTs at strategic geographical locations in the country would provide reliable evidence for health planning and future policy reviews.

## Authors' contributions

Martin Meremikwu, Emmanuel Ezedinachi and Donald Ordu developed the protocol and supervised the trial. All the authors contributed to the preparation of the paper.

**Figure 4:**
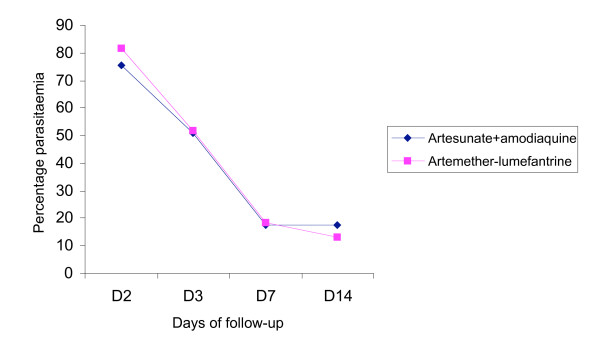
Parasite clearance pattern up to Day-14.
